# Post-Anesthesia Care Unit Duration After Total Hip Arthroplasty Under Spinal Anesthesia in Routine Hospital Practice: A Comparison with Contemporary Arthroplasty Literature

**DOI:** 10.3390/medsci14030397

**Published:** 2026-07-16

**Authors:** Christina Soerensen, Christina Froeslev-Friis, Lisbeth Quitzau, Gunhild Kjaergaard-Andersen, Artur Jawor, Rajesh Prabhakar Bhavsar

**Affiliations:** 1 Department of Anaesthesia and Intensive Care, South Jutland Hospital, 6200 Aabenraa, Denmark; christina.borgen.sorensen@rsyd.dk (C.S.); lisbeth.holmgaard.quitzau2@rsyd.dk (L.Q.); gunhild.kjaergaard-andersen2@rsyd.dk (G.K.-A.); artur.jan.jawor@rsyd.dk (A.J.); 2 Department of Regional Research, University of Southern Denmark, 5230 Aabenraa, Denmark; christina.froeslev-friis2@rsyd.dk

**Keywords:** total hip arthroplasty, spinal anesthesia, post-anesthesia care unit, PACU duration, recovery duration, contextual literature review, perioperative workflow

## Abstract

Background: Post-anesthesia care unit (PACU) duration after total hip arthroplasty (THA) is relevant for ward transfer, early mobilization, and perioperative flow, but is inconsistently reported as a distinct recovery outcome. This study evaluated PACU duration after primary THA under spinal anesthesia in routine hospital-based practice and interpreted the observed duration in relation to contemporary arthroplasty literature. Methods: This retrospective observational cohort study included adult patients undergoing primary THA under spinal anesthesia within two units of a regional hospital. PACU duration was defined as time from PACU arrival until discharge to the orthopedic ward. Demographic, anesthetic, perioperative, and recovery variables were extracted from electronic medical records. Univariate analyses and multivariable linear regression were used to explore factors associated with PACU duration. A focused contextual literature comparison was performed to interpret the observed duration in relation to reported early recovery endpoints, with direct comparison anchored to conventional PACU or recovery-room intervals before ward transfer or immediate pathway progression. Results: Ninety-seven of 150 screened patients were included. Mean PACU duration was 162.3 ± 58.5 min. PACU duration was numerically longer in Unit A than Unit S, but the difference was not statistically significant. Reported early recovery endpoints in the literature were heterogeneous. In univariate analysis, male sex and exploratory spinal local anesthetic dose > 13 mg were associated with longer PACU duration. In multivariable analysis, male sex remained associated with longer PACU duration, whereas spinal dose was not independently associated when analyzed as a continuous variable. Conclusions: PACU duration after THA under spinal anesthesia should be interpreted as a multifactorial, workflow-sensitive outcome rather than a purely anesthetic recovery measure. Differences in discharge criteria, recovery pathways, and endpoint definitions limit direct comparison across studies. PACU duration may be most meaningful as an operational recovery and flow indicator within broadly comparable clinical settings.

## 1. Introduction

Total hip arthroplasty (THA) is a high-volume orthopedic procedure that provides substantial improvement in pain, mobility, functional independence, and health-related quality of life [1,2]. Contemporary THA care is increasingly delivered through standardized perioperative pathways that emphasize multimodal analgesia, early postoperative recovery, mobilization, and coordinated discharge planning [3]. Within these pathways, the immediate postoperative period represents an important transition from surgical completion to active recovery.

Early mobilization is a central component of postoperative recovery after THA [4]. It supports restoration of physical function, improves patient confidence, and may reduce complications related to immobility, including thromboembolic events, pulmonary complications, muscle weakness, delayed functional recovery, and prolonged hospitalization [4]. In many clinical pathways, including the local protocol, structured mobilization is primarily initiated after transfer from the post-anesthesia care unit (PACU) to the orthopedic ward, where nursing and physiotherapy resources are available. PACU duration may therefore influence when active ward-based recovery can begin.

Timely transfer from the PACU, however, must be balanced against patient safety. PACU discharge after THA, particularly after spinal anesthesia, is usually criterion-based rather than time-based [5]. Patients must meet clinical requirements related to physiological stability, oxygenation, pain control, nausea, bleeding status, and regression of spinal anesthesia. Motor recovery is especially relevant after spinal anesthesia because residual motor block may limit safe transfer, standing, and mobilization [6]. PACU duration therefore reflects both recovery from anesthesia and fulfilment of local discharge criteria [7].

PACU duration is also relevant from a perioperative flow perspective [8]. The PACU is a shared clinical resource that must accommodate patients from multiple operating rooms and procedures. Prolonged occupancy may contribute to congestion, delay reception of subsequent postoperative patients, and affect operating-room flow and staffing workload [9]. Conversely, premature transfer may compromise safety if discharge criteria have not been fulfilled [10]. Monitoring PACU duration may therefore help distinguish clinically appropriate recovery from potentially modifiable delays related to patient factors, anesthetic technique, discharge criteria, or local workflow.

Despite its relevance for early mobilization and perioperative flow, PACU duration after THA has not been established as a direct predictor of clinical outcome. Rather, it may be considered an operational and recovery-related indicator because it reflects the time required to achieve physiological stability, adequate symptom control, regression of spinal anesthesia, and readiness for ward transfer. Much of the contemporary THA literature focuses on hospital length of stay, day-of-surgery discharge, complications, readmissions, or functional outcomes [3,11,12]. Studies conducted in the PACU setting often emphasize specific clinical endpoints, such as postoperative nausea and vomiting, pain scores, opioid consumption, or regression of motor block, rather than overall PACU occupancy [6,13,14,15,16]. Consequently, PACU duration remains a relatively underexplored interval in the early postoperative pathway.

These considerations prompted a structured evaluation of PACU duration after THA across two hospital units within the same institutional setting. The pathway had been established for several years in one unit, Unit S, and was more recently introduced in the other, Unit A. This created an opportunity to examine PACU duration across units sharing the same overall institutional operative, anesthetic, and PACU discharge framework.

The aim of this study was to describe PACU duration after primary THA under spinal anesthesia in routine hospital practice, compare PACU duration between the two institutional units, and explore patient-, anesthetic-, and perioperative factors associated with variation in PACU duration. A secondary aim was to interpret the observed local PACU duration in relation to timings reported in contemporary arthroplasty literature through a focused contextual literature comparison. We hypothesized that PACU duration would vary between institutional units and would be associated with patient-, anesthetic-, and perioperative factors, particularly variables related to spinal anesthetic practice.

## 2. Materials and Methods

### 2.1. Study Design and Setting

This retrospective observational cohort study was conducted at the Department of Anesthesiology of a regional hospital consisting of two institutional units. The study included adult patients undergoing primary total hip arthroplasty (THA) between January 2023 and December 2025. Patients were treated within a single hospital system with shared clinical governance and common perioperative recovery standards.

The cohort was analyzed primarily as a unified real-world clinical cohort, while PACU duration was also summarized by institutional unit to describe internal between-unit variation. The study was based on routinely collected perioperative clinical data extracted from electronic patient records, anesthesia charts, and perioperative documentation systems.

As the project was conducted as a retrospective quality and workflow analysis using routinely registered clinical data, formal informed consent was not required according to local institutional practice. Institutional approval for data access and analysis was obtained from the head of the department and the director of the central hospital administration (Reference No. RSYD/12-2023/13456).

This retrospective observational cohort study is reported in accordance with the Strengthening the Reporting of Observational Studies in Epidemiology (STROBE) guidance (Supplementary Materials S3).

### 2.2. Patient Selection and Data Collection

All consecutive adult patients undergoing primary THA during the study period were screened for eligibility. A total of 150 patients was screened. Patients were eligible for inclusion if they underwent primary THA under spinal anesthesia. Patients receiving general anesthesia or combined anesthetic techniques were excluded. Cases with incomplete documentation of PACU arrival time or PACU discharge time were also excluded because the primary outcome could not be determined reliably.

After exclusions, 97 patients undergoing primary THA under spinal anesthesia were included in the final analysis. Analyses were performed as complete-case analyses using patients with available PACU duration data and available covariate information for the planned analyses.

Demographic, anesthetic, perioperative, and recovery-related variables were extracted from electronic anesthesia records and perioperative documentation systems. Collected variables included institutional unit, age, sex, body mass index (BMI), spinal local anesthetic (LA) type, spinal LA dose, vasopressor administration, estimated intraoperative bleeding, and PACU duration.

Spinal anesthetic management reflected routine clinical practice. Spinal LA type was categorized as plain or heavy according to the preparation used. Spinal LA dose was analyzed as a continuous variable in the multivariable regression model. For exploratory univariate analysis, spinal LA dose was additionally categorized as ≤13 mg or >13 mg. This threshold was selected pragmatically based on the observed distribution of spinal LA doses and local dosing practice in the cohort, to distinguish lower-dose from higher-dose spinal anesthetic practice. It was not intended to represent an established or generalizable clinical cut-off. Because this categorization was exploratory, spinal LA dose was analyzed as a continuous variable in the adjusted regression model.

### 2.3. Local Perioperative Pathway

Patients undergoing THA were managed according to a standardized local perioperative pathway incorporating elements consistent with enhanced recovery principles [17]. The standard anesthetic technique for the present cohort was spinal anesthesia with sedation. General anesthesia was used only in selected cases according to local practice, and these patients were not included in the present analysis. No adjuncts were added to the spinal local anesthetic.

Perioperative care included standardized preoperative medication, antibiotic prophylaxis, tranexamic acid when appropriate, multimodal opioid-sparing analgesia with paracetamol and NSAID-based treatment, perioperative glucocorticoid administration where appropriate, surgeon-administered local anesthetic infiltration, structured postoperative monitoring, early assessment of spinal regression and motor recovery, urinary assessment, and criteria-based PACU discharge.

### 2.4. PACU Duration and Discharge Criteria

PACU duration was defined as the time from arrival in the PACU until discharge to the orthopedic ward. PACU discharge was criterion-based rather than time-based and followed routine institutional practice [5]. According to institutional discharge procedures, patients could be discharged by PACU nursing staff when predefined criteria were fulfilled. Patients who did not meet these criteria required review and discharge by an anesthesiologist.

Nurse-led discharge required an uncomplicated surgical, anesthetic, and recovery course, perioperative bleeding below 500 mL, acceptable physiological parameters, and fulfilment of the Danish Society of Anesthesiology and Intensive Care Medicine (DASAIM) recovery score criteria. These criteria included all subscores ≤ 1 and a total score ≤ 4 [5].

For patients recovering after spinal anesthesia, discharge preparation could begin when foot movement had returned. Discharge was permitted when early thigh movement, such as slight knee flexion, was present. Pain was assessed using the numerical rating scale (NRS). Patients were not discharged from the PACU with an NRS score > 4 without prior anesthesiologist contact.

This endpoint reflected routine institutional PACU workflow and criterion-based ward transfer, rather than same-day discharge eligibility, ambulatory discharge readiness, or hospital length of stay.

### 2.5. Contextual Literature Comparison

A focused PubMed search was performed to contextualize the observed PACU duration in relation to contemporary arthroplasty literature. The search focused on adult THA or lower-limb arthroplasty studies reporting PACU duration, recovery-room duration, discharge readiness, motor recovery, or early postoperative pathway timing. The complete search strategy is provided in Supplementary Materials S1.

The search was limited to recent studies in human adult populations and was supplemented by manual screening of relevant references and related articles from key publications. This contextual comparison was not designed as a formal systematic review and was not used for quantitative synthesis. Its purpose was to provide clinical context for interpreting the observed local PACU duration, particularly in relation to differences in recovery endpoints and discharge criteria across studies.

### 2.6. Statistical Analysis

PACU duration was analyzed as a continuous variable. Continuous variables are presented as mean ± standard deviation, and categorical variables as number and percentage. The distribution of PACU duration was assessed by visual inspection of histograms and Q-Q plots and by the Shapiro–Wilk test.

The primary analysis was performed in the overall cohort. PACU duration was also summarized by institutional unit as a descriptive internal practice comparison. For this unit-level comparison, both an independent-samples *t*-test and a Mann–Whitney U test were used. The *t*-test was used to compare mean PACU duration, whereas the Mann–Whitney U test was used as a non-parametric sensitivity analysis because of the modest sample size and evidence of non-normality in the overall cohort. Welch’s *t*-test was also reported as a sensitivity analysis for unequal variance (Supplementary Materials S1 and S2).

Univariate analyses were performed in the combined cohort to explore associations between perioperative variables and PACU duration. Categorical variables, including sex, vasopressor administration, spinal LA type, and exploratory spinal LA dose category, were analyzed using independent-samples *t*-tests. Continuous variables, including age, BMI, estimated bleeding volume, and spinal LA dose in mg, were analyzed using correlation analysis. Because these analyses were exploratory and hypothesis-generating, no formal adjustment for multiple comparisons was applied.

Multivariable linear regression was performed with PACU duration as the dependent variable. Covariates were selected a priori based on clinical plausibility and availability in the retrospective dataset, rather than on statistical significance in univariate analyses. The selected covariates were sex, age, BMI, spinal LA type, spinal LA dose as a continuous variable, vasopressor administration, and estimated bleeding volume. No interaction terms were included because of the modest cohort size and the exploratory purpose of the regression analysis.

Regression diagnostics were performed after model estimation. Model fit was described using R^2^, adjusted R^2^, the overall F-test, and root mean squared error (RMSE). Residual distribution was assessed by visual inspection and by the Shapiro–Wilk test. Homoscedasticity was assessed using residual plots and the Breusch–Pagan test. Autocorrelation was assessed using the Durbin–Watson statistic. Influential observations were assessed using studentized residuals and Cook’s distance. Multicollinearity was assessed using variance inflation factors (VIFs). Because spinal LA type was strongly associated with institutional unit, collinearity between unit and LA type was also explored as part of model interpretation.

Statistical significance was defined as a two-sided *p*-value < 0.05. Statistical analyses were performed using Stata/BE version 19.0 (Stata Corp LLC., College Station, TX, USA).

## 3. Results

**Figure 1:**
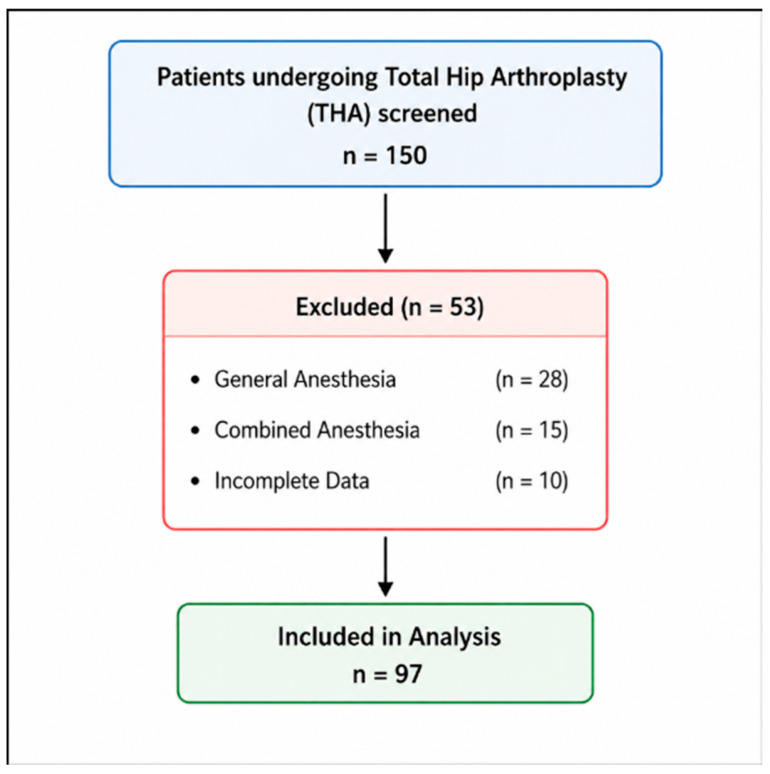
Flowchart illustrating patient selection and cohort formation. A total of 150 adult patients undergoing primary total hip arthroplasty (THA) during the study period were screened for eligibility. Patients receiving general anesthesia, combined anesthetic techniques, or with incomplete PACU-duration documentation were excluded. A total of 97 patients undergoing THA under spinal anesthesia were included in the final complete-case analysis. PACU = post-anesthesia care unit.

A total of 150 patients undergoing primary THA was screened during the study period. After exclusion of patients receiving general anesthesia, combined anesthetic techniques, or incomplete PACU-duration documentation, 97 patients undergoing primary THA under spinal anesthesia were included in the final analysis (Figure 1).

Baseline demographic, anesthetic, and perioperative characteristics are summarized in Table 1. The cohort included 47 patients from Unit A and 50 patients from Unit S. Mean overall PACU duration was 162.3 ± 58.5 min.

As a secondary descriptive observation, PACU duration was summarized according to institutional unit. PACU duration showed some deviation from normality in the overall cohort by Shapiro–Wilk testing (W = 0.964, *p* = 0.0085), although unit-specific distributions did not show statistically significant deviation from normality (Unit A, *p* = 0.0679; Unit S, *p* = 0.0705). Mean PACU duration was 171.7 ± 63.6 min in Unit A and 153.5 ± 52.2 min in Unit S. This corresponded to a mean difference of 18.2 min. The difference was not statistically significant using an independent-samples *t*-test (*p* = 0.125), Welch’s *t*-test (*p* = 0.128), or Mann–Whitney U test (*p* = 0.209). The Mann–Whitney U test was included as a non-parametric sensitivity analysis because of the modest sample size and non-normality in the overall cohort (Supplementary Materials S2).

### Factors Associated with PACU Duration

Univariate analysis of the combined cohort showed longer PACU duration in male patients (+30.6 min, *p* = 0.009). PACU duration was also longer in patients receiving spinal LA doses > 13 mg (+24.5 min, *p* = 0.042). This dose category was exploratory and was used only to describe lower- versus higher-dose spinal anesthetic practice within the observed cohort. No statistically significant associations were identified for spinal LA type, estimated bleeding volume, age, vasopressor administration, or BMI (Table 2).

In multivariable linear regression analysis of the combined cohort, male sex remained associated with longer PACU duration (β = +28.8 min, 95% CI 3.8 to 53.9, *p* = 0.025). No other covariates, including spinal LA type or spinal LA dose analyzed as a continuous variable, demonstrated a statistically significant independent association with PACU duration (Table 3).

The overall regression model explained a modest proportion of variation in PACU duration (R^2^ = 0.110, adjusted R^2^ = 0.040; F = 1.57, *p* = 0.156; RMSE = 57.3 min). Residual diagnostics did not indicate major heteroscedasticity or influential observations. The Breusch–Pagan test was not statistically significant (*p* = 0.222), no studentized residual exceeded 3, and the maximum Cook’s distance was 0.093. Residuals showed mild deviation from normality by Shapiro–Wilk testing (*p* = 0.0439). Variance inflation factors in the final model were low, with all VIF values ≤ 1.21.

Although variance inflation factors were low in the final regression model, spinal LA type differed markedly between institutional units. Therefore, the regression coefficient for spinal LA type should be interpreted cautiously and should not be considered an isolated pharmacological effect independent of unit-related practice patterns.

The contextual literature comparison is presented in the Discussion to support interpretation of the observed PACU duration in relation to comparable PACU-to-ward transfer endpoints and broader, non-equivalent discharge-readiness endpoints.

## 4. Discussion

The principal finding of this study was that PACU duration following primary THA under spinal anesthesia in routine hospital-based practice was longer than many conventional PACU or recovery-room intervals reported in contemporary arthroplasty literature. Despite marked differences in spinal anesthetic practice, PACU duration did not differ significantly between the two institutional units. In exploratory analyses of the combined cohort, male sex remained independently associated with longer PACU duration, whereas higher spinal local anesthetic dose was associated with longer duration only in univariate analysis. Taken together, these findings support the interpretation of PACU duration as a multifactorial and workflow-sensitive perioperative interval rather than as a purely anesthetic recovery measure.

A relevant observation from the present study is the difference between PACU duration in routine clinical practice and the shorter recovery intervals reported in selected contemporary arthroplasty studies. This comparison should be anchored primarily to studies reporting conventional PACU or recovery-room duration before ward transfer or immediate pathway progression, because these endpoints most closely resemble the present outcome. Modern THA pathways based on enhanced recovery principles emphasize early mobilization, functional recovery, reduced length of stay, and, in selected settings, same-day discharge. Several studies using low-dose or short-acting spinal anesthetic strategies have reported faster motor recovery and earlier ambulation. However, these studies often originate from highly protocolized pathways, selected patient populations, and anesthetic regimens specifically designed to facilitate rapid recovery. In contrast, PACU duration in routine hospital-based practice may be influenced not only by regression of neuraxial blockade, but also by discharge criteria, mobilization timing, bladder assessment, pain control, nausea, physiotherapy availability, ward-bed availability, and local transfer logistics.

The contextual literature comparison further showed that published early recovery endpoints after THA vary considerably. PACU discharge, PACU duration, motor recovery, ambulation, discharge readiness, and full facility discharge are not equivalent outcomes. Most identified studies compared recovery outcomes between interventions such as spinal anesthetic agents, anesthetic technique, spinal dose strategies, intrathecal opioid use, or PACU discharge pathways, and only one study used historical PACU duration as a comparator [18]. Among studies reporting conventional PACU or immediate recovery-room duration before ward transfer or early pathway progression, reported durations ranged from approximately 70 to 154 min, with other reported intervals including 93–96 min, 76–114 min, and 108 min [19,20,21,22,23]. In contrast, ambulatory or outpatient arthroplasty pathways reported longer recovery intervals, including PACU/recovery durations of 4.0 to 5.7 h and mean discharge readiness of approximately 359 min, reflecting discharge-home readiness rather than ward transfer [24,25]. These broader endpoints incorporated functional requirements such as ambulation, voiding, pain control, stair ability, physiotherapy clearance, and readiness for home discharge. Therefore, the PACU duration observed in the present cohort should be interpreted in relation to the specific endpoint measured, namely time from PACU arrival until discharge to the orthopedic ward, and the routine hospital-based setting in which recovery occurred.

Comparison between the two institutional units was included to assess whether PACU duration differed within the same overall institutional operative, anesthetic, and PACU discharge framework. PACU duration was numerically longer in Unit A than in Unit S, but the difference did not reach statistical significance. Interpretation of this comparison is limited by the marked imbalance in spinal LA type between units. Unit A predominantly used plain/isobaric spinal LA, whereas Unit S predominantly used heavy/hyperbaric spinal LA. [26] Hospital unit and LA type were therefore strongly associated, and the present data cannot reliably separate local anesthetic effects from unit-related workflow or practice effects. Accordingly, the LA-type coefficient in the regression model should not be interpreted as an independent pharmacological effect. The absence of a statistically significant unit difference suggests that prolonged PACU duration was not confined to one unit, but more likely reflected broader patient-, pathway-, and workflow-related factors across the institutional settings.

In the exploratory analyses, male sex and higher spinal LA dose showed different patterns after adjustment. Male sex remained independently associated with longer PACU duration in the multivariable model, suggesting that this association was not fully explained by age, BMI, spinal LA type, spinal dose, bleeding, or vasopressor use. The mechanism underlying this association cannot be determined from the present data. It may relate to unmeasured postoperative or workflow-related factors such as bladder assessment, delayed voiding, motor block regression, pain, nausea, mobilization readiness, or fulfilment of discharge criteria. Postoperative urinary retention is one possible explanation, as urinary retention and delayed voiding have been reported as barriers to discharge in arthroplasty pathways [8,9]. However, bladder function, bladder scanning, catheterization, and time to voiding were not systematically captured in the present dataset. Therefore, urinary retention should be regarded as a hypothesis rather than a demonstrated mechanism.

In contrast, the univariate association between spinal LA dose > 13 mg and prolonged PACU duration was not retained when spinal LA dose was analyzed as a continuous variable in the multivariable model. The 13 mg threshold was used as an exploratory pragmatic categorization to compare lower- and higher-dose spinal anesthetic practice within the observed cohort. It was not intended to represent an established clinical threshold. The loss of association after adjustment suggests that the univariate finding may reflect local practice patterns, spinal LA type, non-linear dose effects, or residual confounding rather than a simple independent dose–response relationship. From a clinical perspective, spinal LA dose remains relevant because anesthetic dosing in fast-track arthroplasty must balance intraoperative reliability against postoperative recovery efficiency [27]. Lower doses or shorter-acting spinal agents may facilitate earlier motor recovery and mobilization [6], but the present findings do not establish an optimal spinal dose. Therefore, higher spinal LA dose should be interpreted as a hypothesis-generating and potentially modifiable factor rather than an independent predictor of prolonged PACU duration.

No significant independent associations were observed between PACU duration and estimated bleeding, vasopressor administration, age, BMI, spinal LA type, or spinal LA dose. These findings suggest that, within the variables available in this dataset, PACU duration was not primarily explained by measured indicators of perioperative bleeding, hemodynamic support, or demographic factors other than sex. The multivariable model explained only a modest proportion of the variation in PACU duration, supporting the likelihood that unmeasured clinical and organizational factors contributed substantially. These may include motor block regression, bladder function, pain trajectory, nausea, dizziness, physiotherapy timing, ward-bed availability, and specific reasons for delayed discharge.

Overall, the findings suggest that PACU duration after THA under spinal anesthesia should be interpreted as a distinct perioperative interval that integrates anesthetic recovery, functional readiness, discharge criteria, and local workflow. Although the present study cannot establish PACU duration as a direct predictor of complications, delayed mobilization, or hospital length of stay, it supports its relevance as a practical recovery and flow indicator within comparable hospital-based arthroplasty pathways. Future prospective studies should capture motor recovery, bladder function, mobilization timing, pain and nausea scores, discharge criteria fulfilment, and ward-transfer logistics to better define the determinants and clinical consequences of prolonged PACU duration after THA.

### Limitations

This study has limitations. The retrospective observational design limits causal interpretation, and the modest cohort size relative to the number of covariates may have reduced statistical power and precision in the multivariable model. The analysis was performed as a complete-case analysis, and patients with incomplete PACU arrival or discharge time documentation were excluded because the primary outcome could not be reliably determined. Although regression diagnostics did not indicate major heteroscedasticity or extreme influential outliers, the multivariable model explained only a modest proportion of the variation in PACU duration. No interaction terms were included because of the exploratory nature of the analysis and the limited sample size.

Several clinically relevant recovery variables were not routinely documented. These included detailed motor block regression, bladder function, bladder scanning, catheterization, time to voiding, time to mobilization, pain trajectory, nausea, dizziness, physiotherapy timing, ward-bed availability, and specific reasons for delayed PACU discharge. Therefore, some explanations for prolonged PACU duration, including the possible role of urinary retention or delayed mobilization readiness, remain inferential.

Anesthetic practice differed between the two institutional units, particularly regarding spinal LA type. This created substantial confounding between hospital unit and anesthetic technique and limits the ability to separate site-related workflow effects from local anesthetic effects. Accordingly, the LA-type coefficient should not be interpreted as an independent pharmacological effect. Similarly, the >13 mg spinal LA dose category was used only as an exploratory pragmatic categorization within the observed cohort and does not represent an established clinical threshold.

The contextual literature comparison was intended only to provide contemporary clinical context and was not designed as a formal systematic review or quantitative synthesis. Published early recovery endpoints varied considerably between studies, including PACU duration, recovery-room duration, motor recovery, discharge readiness, and full facility discharge. Direct comparison with the present cohort is therefore limited and should be anchored primarily to studies reporting conventional PACU or recovery-room intervals before ward transfer or immediate pathway progression.

Despite these limitations, the study reflects routine clinical practice and addresses an operationally important perioperative interval that is inconsistently reported in contemporary THA anesthesia literature. The findings should be interpreted as hypothesis-generating and as a basis for future prospective studies with more detailed capture of recovery, discharge, and workflow variables.

## 5. Conclusions

PACU duration after primary THA under spinal anesthesia is a multifactorial and workflow-sensitive outcome, not merely a measure of anesthetic recovery. Although the observed duration was longer than many conventional PACU or recovery-room intervals reported before ward transfer or immediate pathway progression, differences in discharge criteria, recovery pathways, and endpoint definitions limit direct comparison across studies. PACU duration may be most meaningful as an operational recovery and flow indicator within broadly comparable clinical settings.

## Figures and Tables

**Table 1:** Baseline Demographic and Perioperative Characteristics of the Study Cohort.

Variable	Overall (n = 97)	Unit A (n = 47)	Unit S (n = 50)
Age, years	69.3 ± 10.7	71.1 ± 10.0	67.6 ± 11.2
Male sex, n (%)	44 (45.4%)	20 (42.6%)	24 (48.0%)
BMI, kg/m^2^	28.3 ± 5.4	28.7 ± 5.6	27.8 ± 5.3
Heavy spinal LA, n (%)	49 (50.5%)	4 (8.5%)	45 (90.0%)
High spinal LA dose (>13 mg), n (%)	39 (40.2%)	16 (34.0%)	23 (46.0%)
Vasopressor administration, n (%)	93 (95.9%)	47 (100.0%)	46 (92.0%)
Estimated bleeding, mL	272 ± 124	257 ± 121	286 ± 127
PACU duration, min	162.3 ± 58.5	171.7 ± 63.6	153.5 ± 52.2

Baseline demographic, anesthetic, and perioperative characteristics of patients undergoing primary total hip arthroplasty under spinal anesthesia at the two participating institutional units. Data are presented as mean ± standard deviation or number (%), as appropriate. PACU duration represents total time spent in the post-anesthesia care unit from arrival until discharge to the orthopedic ward. Spinal LA dose > 13 mg was used as an exploratory pragmatic categorization to describe lower- versus higher-dose spinal anesthetic practice within the observed cohort and was not intended to represent an established clinical threshold. BMI = body mass index; LA = local anesthetic; PACU = post-anesthesia care unit.

**Table 2:** Univariate analysis of factors associated with postoperative recovery duration.

Variable	Comparison/Analysis	Effect on Recovery Duration	*p*-Value
Sex	Male vs. Female	+30.6 min in males	0.009 *
Spinal LA dose	>13 mg vs. ≤13 mg	+24.5 min with higher dose	0.042 *
Spinal LA type	Plain vs. Heavy	+18.7 min with plain LA	0.130 *
Bleeding	Continuous variable	r = −0.17	0.102 **
Age	Continuous variable	r = −0.11	0.282 **
Vasopressor use	Yes vs. No	−3.4 min	0.790 *
BMI	Continuous variable	r = 0.02	0.827 **

Univariate analysis of perioperative factors associated with PACU duration. PACU duration was defined as time from arrival in the post-anesthesia care unit until discharge to the orthopedic ward. Positive values indicate longer PACU duration, while negative values indicate shorter PACU duration. The spinal LA dose category of >13 mg was exploratory and was used to describe lower- versus higher-dose spinal anesthetic practice within the observed cohort; spinal LA dose was analyzed as a continuous variable in the multivariable regression model. * Independent-samples *t*-test. ** Pearson correlation. LA = local anesthetic; BMI = body mass index; PACU = post-anesthesia care unit.

**Table 3:** Multivariable linear regression analysis of factors associated with PACU duration.

Variable	Regression Coefficient (β), min	95% CI	*p*-Value
Male sex	+28.8	3.8 to 53.9	0.025
Age (per year)	−0.65	−1.82 to 0.53	0.276
Estimated bleeding (per mL)	−0.05	−0.15 to 0.06	0.362
Vasopressor use	+9.4	−18.0 to 36.7	0.498
Heavy spinal LA type *	−17.3	−41.1 to 6.4	0.151
Spinal LA dose (per mg)	−0.76	−8.2 to 6.7	0.839
BMI (per kg/m^2^)	+0.33	−1.93 to 2.60	0.770

PACU duration was analyzed as a continuous dependent variable, defined as time from arrival in the post-anesthesia care unit until discharge to the orthopedic ward. Positive coefficients indicate longer PACU duration. * Heavy spinal LA type was strongly associated with institutional unit and should therefore be interpreted cautiously. CI = confidence interval; BMI = body mass index; LA = local anesthetic; PACU = post-anesthesia care unit.

## Data Availability

The data underlying this study were derived from routine electronic medical records and are not publicly available due to institutional data-protection regulations and patient confidentiality. Anonymized aggregated data may be made available from the corresponding author upon reasonable request and subject to institutional approval.

## Supplementary Materials

The following supporting information can be downloaded at: https://www.mdpi.com/article/10.3390/medsci14030397/s1, Supplementary Material S1: PubMed search strategy for the focused contextual literature comparison. This file provides the PubMed search date, search terms, filters, supplementary screening approach, and purpose of the contextual literature search used to identify contemporary arthroplasty studies reporting PACU, recovery-room, discharge-readiness, or early postoperative recovery outcomes. Supplementary Material S2: Comparison of PACU duration between the two participating institutional units. This file summarizes mean and median PACU duration, standard deviation, sample size, between-unit differences, and statistical comparison between Unit A and Unit S. Supplementary Material S3: STROBE checklist for the THAPACU retrospective observational cohort study. This file provides the completed STROBE cohort-study reporting checklist, including manuscript page references and reporting status for each checklist item.

## Abbreviations

Abbreviation	Definition
BMI	Body mass index
CI	Confidence interval
DASAIM	Danish Society of Anesthesiology and Intensive Care Medicine
ERAS	Enhanced Recovery After Surgery
LA	Local anesthetic
NRS	Numerical rating scale
NSAID	Non-steroidal anti-inflammatory drug
PACU	Post-anesthesia care unit
SD	Standard deviation
THA	Total hip arthroplasty
