# Supplementary material for: Post-Anesthesia Care Unit Duration After Total Hip Arthroplasty Under Spinal Anesthesia in Routine Hospital Practice: A Comparison with Contemporary Arthroplasty Literature
Source: Med Sci (Basel). 2026 Jul 16;14(3):397. doi: 10.3390/medsci14030397 (PMC13413505; doi:10.3390/medsci14030397)
Supplement: Supplementary file 1 [file medsci-14-00397-s001.zip › Supplementary Materials S1 and S2.pdf]

Supplementary Material S1

PubMed search strategy for focused contextual literature review

Search date: 26/04/2026

A focused contextual literature search was performed in PubMed to identify contemporary studies reporting post-anesthesia care unit (PACU), recovery-room, or discharge-readiness outcomes after total hip arthroplasty, particularly in relation to spinal anesthesia and early postoperative recovery. The search was intended to contextualize the observed PACU duration and was not designed as a systematic review.

Database searched

PubMed

Search strategy

```
("total hip arthroplasty"[Title/Abstract] OR "total hip replacement"[Title/Abstract] OR
THA[Title/Abstract] OR "hip arthroplasty"[Title/Abstract])
AND
("spinal anesthesia"[Title/Abstract] OR "spinal anaesthesia"[Title/Abstract] OR
neuraxial[Title/Abstract] OR "subarachnoid block"[Title/Abstract])
AND
(PACU[Title/Abstract] OR "post-anesthesia care unit"[Title/Abstract] OR "post-
anaesthesia care unit"[Title/Abstract] OR "recovery room"[Title/Abstract] OR
"postoperative recovery"[Title/Abstract] OR "discharge readiness"[Title/Abstract] OR
"same-day discharge"[Title/Abstract])
```

Filters applied

- English language
- Adult humans
- Publication years 2020-2026

Supplementary screening

The search was supplemented by screening titles, abstracts, and reference lists of relevant articles to identify additional studies reporting early recovery, PACU duration, recovery-room duration, discharge readiness, mobilization, urinary retention, same-day discharge, or perioperative flow outcomes in contemporary hip or lower-limb arthroplasty pathways.

Purpose and scope

Studies were selected based on relevance to contextual interpretation of PACU duration after THA under spinal anesthesia. The aim was to compare the observed institutional recovery duration with reported PACU, recovery-room, and discharge-readiness intervals in contemporary arthroplasty literature, rather than to perform a formal systematic review or meta-analysis.

Abbreviations: PACU = post-anesthesia care unit; THA = total hip arthroplasty.

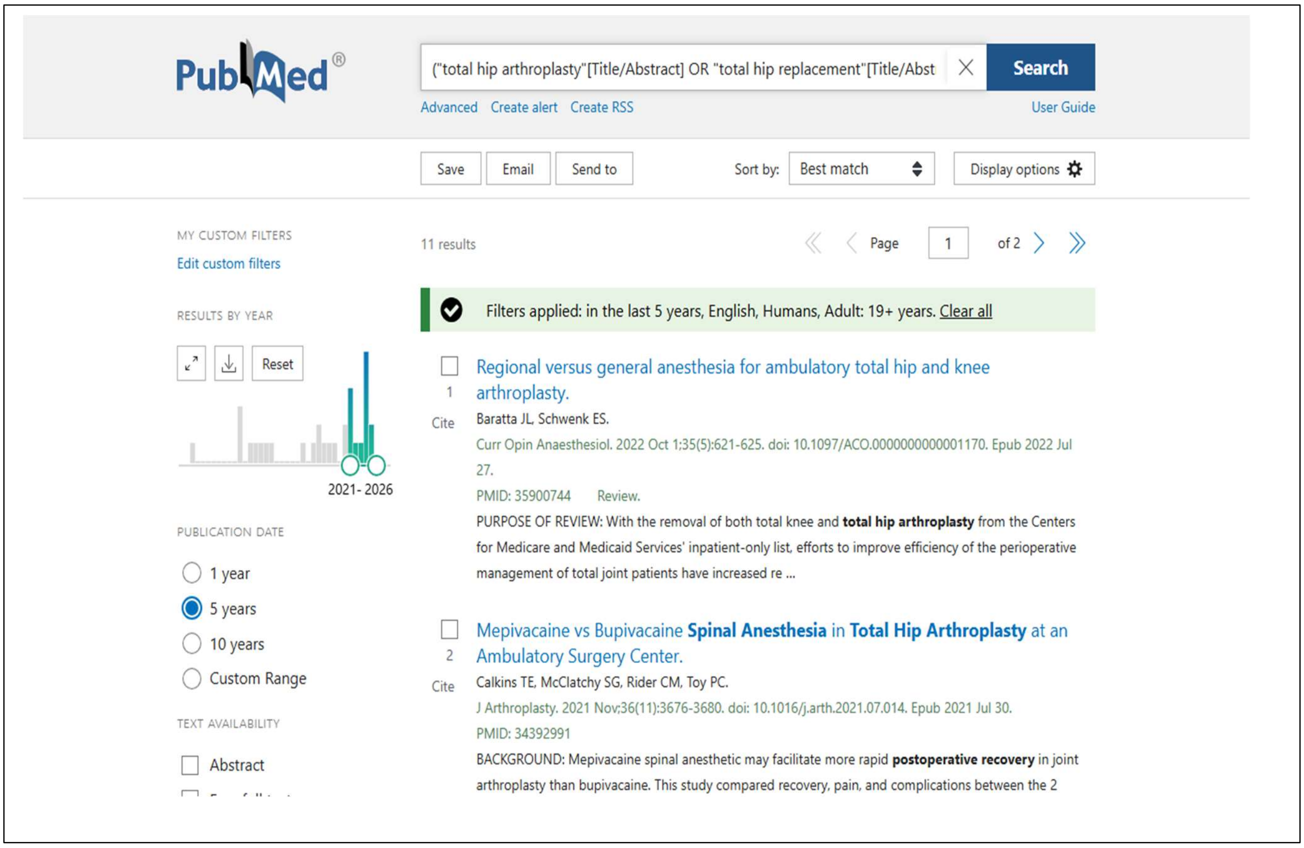

Supplementary Material S2. Comparison PACU duration between the two participating units

Unit	Mean PACU Duration (min)	Median PACU Duration (min)	Standard Deviation (min)	n
Unit A	171.7	163.0	63.6	47
Unit S	153.5	148.5	52.2	50
Mean / Median Difference	+18.2	+14.5	–	–

Comparison of PACU duration between the two participating institutional units. PACU duration was defined as total time spent in the PACU from arrival until discharge to the orthopedic ward. Although mean duration was longer in Unit A compared with Unit S, the difference did not reach statistical significance using either parametric or non-parametric testing. Independent-samples t-test; $p = 0.125$; Mann–Whitney U test: $p = 0.209$. PACU= Post anesthesia care unit
