# Supplementary material for: Post-Anesthesia Care Unit Duration After Total Hip Arthroplasty Under Spinal Anesthesia in Routine Hospital Practice: A Comparison with Contemporary Arthroplasty Literature
Source: Med Sci (Basel). 2026 Jul 16;14(3):397. doi: 10.3390/medsci14030397 (PMC13413505; doi:10.3390/medsci14030397)
Supplement: Supplementary file 1 [file medsci-14-00397-s001.zip › Supplementary_Material_S3 _STROBE_Checklist_THAPACU.pdf]

Supplementary Material - STROBE Checklist - THAPACU

STROBE Statement - Checklist for Cohort Studies

THAPACU: Post-anesthesia care unit duration after total hip arthroplasty under spinal anesthesia in routine hospital practice

Supplementary Material: Reporting checklist

Manuscript version checked: V2-THAPACU-V2.1.docx, clean revised manuscript. Study design: retrospective observational cohort / two-unit comparison. Prepared: 9 July 2026.

Note: The first column contains the official STROBE item number. The dedicated "Manuscript page(s)" column gives the page reference in the checked Word/PDF rendering. Page references are approximate and may change if the manuscript formatting changes. This checklist is intended for upload as supplementary material or as a reporting checklist, not as a section in the main manuscript.

STROBE item no.	Section / topic	STROBE recommendation	Manuscript page(s)	Where reported / status
1(a)	Title and abstract	Indicate the study design with a commonly used term in the title or abstract.	p. 1	Title/Abstract. Abstract states retrospective observational cohort study. Reported.
1(b)	Title and abstract	Provide an informative and balanced summary of what was done and what was found.	p. 1	Abstract summarizes background, methods, results and conclusion, including cohort size, PACU duration, unit comparison and regression findings. Reported.
2	Introduction - Background/rationale	Explain the scientific background and rationale for the investigation.	pp. 2-3	Rationale addresses PACU duration, early mobilisation, criterion-based discharge, perioperative flow and under-reporting of PACU duration. Reported.
3	Introduction - Objectives	State specific objectives, including any prespecified hypotheses.	p. 3	Objectives and hypothesis are stated: describe PACU duration, compare units, explore associated factors and contextualize against arthroplasty literature. Reported.
4	Methods - Study design	Present key elements of study design early in the paper.	p. 3	Methods 2.1 states retrospective observational cohort study. Reported.
5	Methods - Setting	Describe the setting, locations and relevant dates, including periods of recruitment, exposure, follow-up and data collection.	p. 3	Regional hospital with two institutional units; primary THA between January 2023 and December 2025; routine perioperative data. Reported.
6(a)	Methods - Participants	Give eligibility criteria and the sources and methods of selection of participants. Describe methods of follow-up for cohort studies.	p. 4	Methods 2.2 describes consecutive adult primary THA patients screened; spinal anesthesia required; general/combined anesthesia and incomplete PACU-time documentation excluded. No follow-up beyond PACU discharge outcome. Reported.
6(b)	Methods - Participants	For matched studies, give matching criteria and number of exposed and unexposed.	N/A	Not applicable; this was not a matched cohort study.
7	Methods - Variables	Clearly define all outcomes, exposures, predictors, potential confounders and effect modifiers.	pp. 4-5	Methods 2.2-2.4 define institutional unit, age, sex, BMI, spinal LA type/dose, vasopressor use, bleeding and PACU duration. PACU duration is PACU arrival to discharge to orthopedic ward. Reported.

STROBE item no.	Section / topic	STROBE recommendation	Manuscript page(s)	Where reported / status
8	Methods - Data sources/measurement	For each variable of interest, give sources of data and details of methods of assessment/measurement. Describe comparability if more than one group.	pp. 3-5	Data extracted from electronic patient records, anesthesia charts and perioperative documentation systems. Common institutional pathway and PACU discharge criteria described. Reported.
9	Methods - Bias	Describe any efforts to address potential sources of bias.	pp. 4-6; pp. 12-13	Consecutive screening, exclusion of unreliable PACU-time documentation, complete-case approach, diagnostics and cautious interpretation of collinearity/unmeasured variables are described. Reported, with limitations acknowledged.
10	Methods - Study size	Explain how the study size was arrived at.	pp. 4 and 6-7	All consecutive eligible patients during study period were screened; 150 screened and 97 included. Retrospective consecutive cohort; no formal sample-size calculation required. Reported.
11	Methods - Quantitative variables	Explain how quantitative variables were handled in the analyses. If applicable, describe which groupings were chosen and why.	pp. 4-6	PACU duration analyzed continuously; spinal LA dose analyzed continuously in regression and categorized at <=13 mg/>13 mg only for exploratory univariate analysis with rationale. Reported.
12(a)	Methods - Statistical methods	Describe all statistical methods, including those used to control for confounding.	pp. 5-6	Descriptive statistics, t-test, Mann-Whitney U, Welch sensitivity analysis, univariate analyses and multivariable linear regression with clinically selected covariates are described. Reported.
12(b)	Methods - Statistical methods	Describe any methods used to examine subgroups and interactions.	p. 6	Unit-level comparison described; no interaction terms included because of modest cohort size and exploratory purpose. Reported; interaction analysis not performed and rationale provided.
12(c)	Methods - Statistical methods	Explain how missing data were addressed.	p. 4	Incomplete PACU arrival/discharge documentation was excluded; analyses performed as complete-case analyses. Reported.
12(d)	Methods - Statistical methods	If applicable, explain how loss to follow-up was addressed in cohort studies.	N/A	Not applicable; the outcome was derived from the same PACU episode and no longitudinal follow-up was used.
12(e)	Methods - Statistical methods	Describe any sensitivity analyses.	pp. 5 and 8	Mann-Whitney U and Welch t-test reported as sensitivity analyses for the unit-level comparison. Reported.
13(a)	Results - Participants	Report numbers of individuals at each stage of study: eligible, included, completed follow-up and analyzed.	pp. 6-7	Results and Figure 1 report 150 screened, 53 excluded and 97 included in the final complete-case analysis. Reported.
13(b)	Results - Participants	Give reasons for non-participation at each stage.	p. 7	Figure 1 gives exclusion reasons: general anesthesia, combined anesthesia and incomplete data. Reported.
13(c)	Results - Participants	Consider use of a flow diagram.	p. 7	Figure 1 provides the flow diagram. Reported.
14(a)	Results - Descriptive data	Give characteristics of study participants and information on exposures and potential confounders.	p. 7	Table 1 reports baseline demographic, anesthetic and perioperative characteristics for the overall cohort and by unit. Reported.

STROBE item no.	Section / topic	STROBE recommendation	Manuscript page(s)	Where reported / status
14(b)	Results - Descriptive data	Indicate number of participants with missing data for each variable of interest.	pp. 4 and 7	Incomplete PACU-duration documentation is reported as an exclusion category and complete-case analysis is stated. Partly reported; consider adding a short table note confirming covariate-level missingness among included patients if required by the journal.
14(c)	Results - Descriptive data	Summarize follow-up time for cohort studies.	N/A	Not applicable; no longitudinal follow-up. Outcome was PACU duration during the index postoperative episode.
15	Results - Outcome data	Report numbers of outcome events or summary measures over time.	pp. 7-8	Mean overall PACU duration 162.3 +/- 58.5 minutes; unit-specific PACU duration 171.7 +/- 63.6 vs 153.5 +/- 52.2 minutes. Reported.
16(a)	Results - Main results	Give unadjusted estimates and, if applicable, confounder-adjusted estimates and their precision.	pp. 8-9	Tables 2 and 3 report univariate associations and multivariable regression coefficients with 95% CIs and p-values. Reported.
16(b)	Results - Main results	Report category boundaries when continuous variables are categorized.	pp. 4 and 7-8	Spinal LA dose category <=13 mg vs >13 mg is defined and described as exploratory. Reported.
16(c)	Results - Main results	If relevant, consider translating relative risk into absolute risk for a meaningful time period.	N/A	Not applicable; outcome is continuous PACU duration, not a binary risk.
17	Results - Other analyses	Report other analyses done, such as subgroup analyses, interaction analyses and sensitivity analyses.	pp. 8-9	Unit-level comparison, sensitivity tests, regression diagnostics and model-fit statistics are reported. Reported.
18	Discussion - Key results	Summarize key results with reference to study objectives.	p. 10	Discussion summarizes PACU duration, unit comparison, male sex association and spinal-dose finding. Reported.
19	Discussion - Limitations	Discuss limitations, taking into account sources of potential bias or imprecision.	pp. 12-13	Limitations discuss retrospective design, modest cohort size, complete-case analysis, unmeasured variables, collinearity and contextual-review limitations. Reported.
20	Discussion - Interpretation	Give a cautious overall interpretation considering objectives, limitations, multiplicity and evidence from similar studies.	pp. 10-12	Interpretation is cautious and separates PACU-to-ward endpoints from broader discharge-home readiness intervals. Reported.
21	Discussion - Generalisability	Discuss generalisability of the results.	pp. 12-13	Findings are interpreted as relevant to comparable routine hospital-based arthroplasty pathways rather than highly optimized ambulatory settings. Reported.
22	Other information - Funding	Give the source of funding and the role of funders for the present study and, if applicable, for the original study on which the article is based.	Declarations / end matter	Funding statement indicates no external funding, provided the declarations section is retained in the submitted manuscript. Reported.

Final internal note: Before final journal submission, confirm that the declarations section and any journal-specific STROBE template requirements are included in the final manuscript package.
